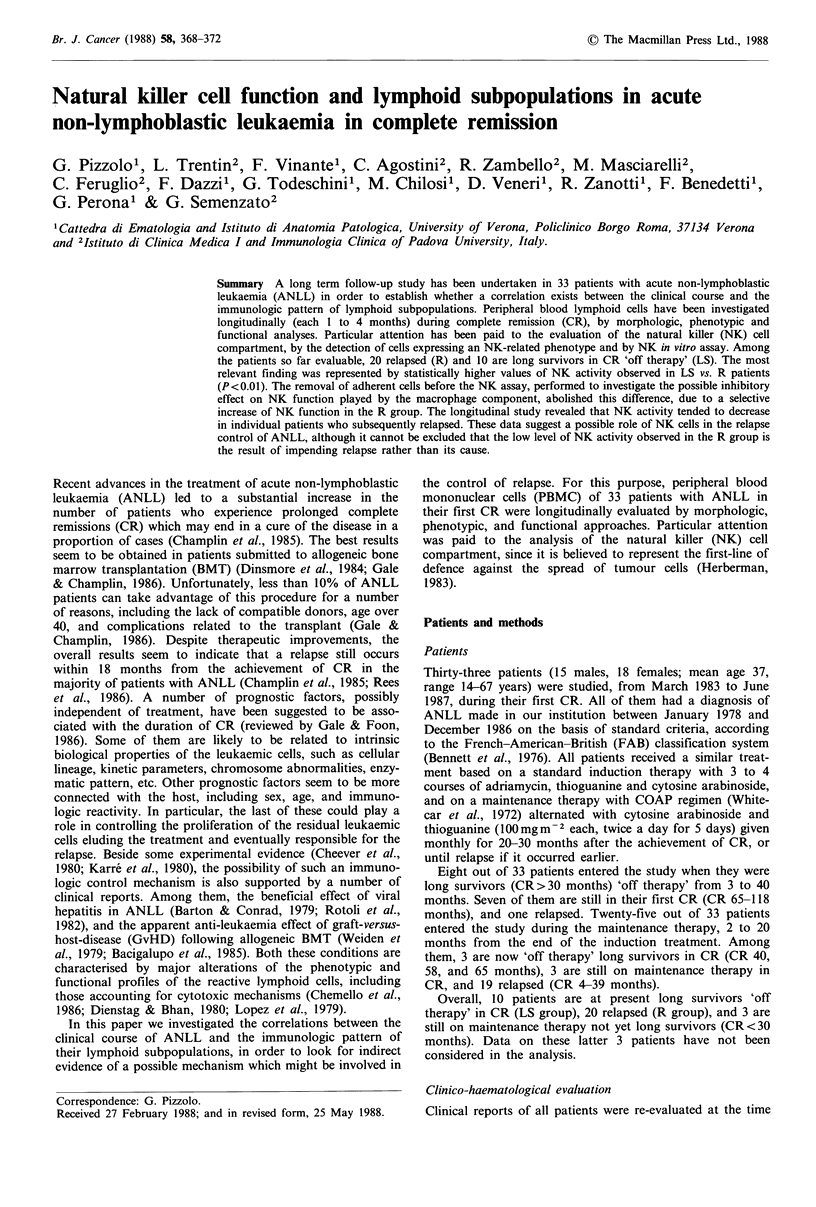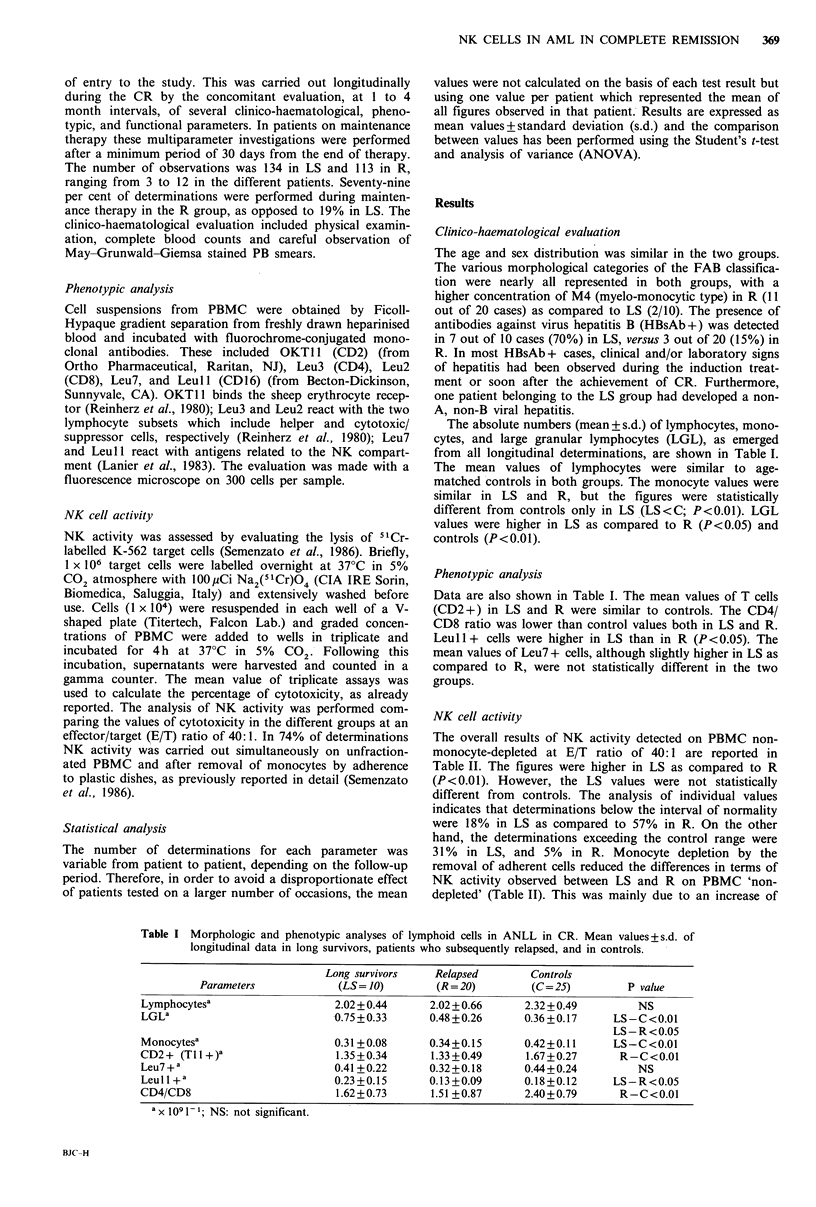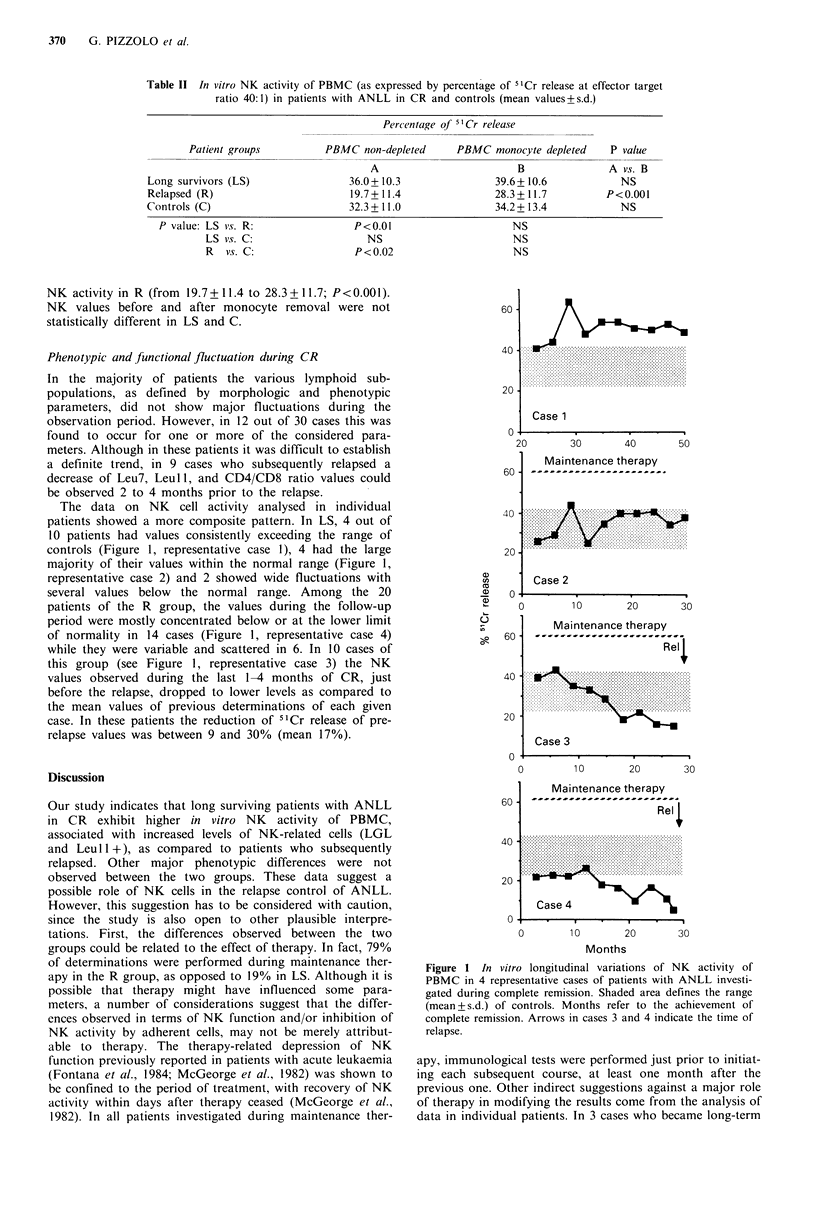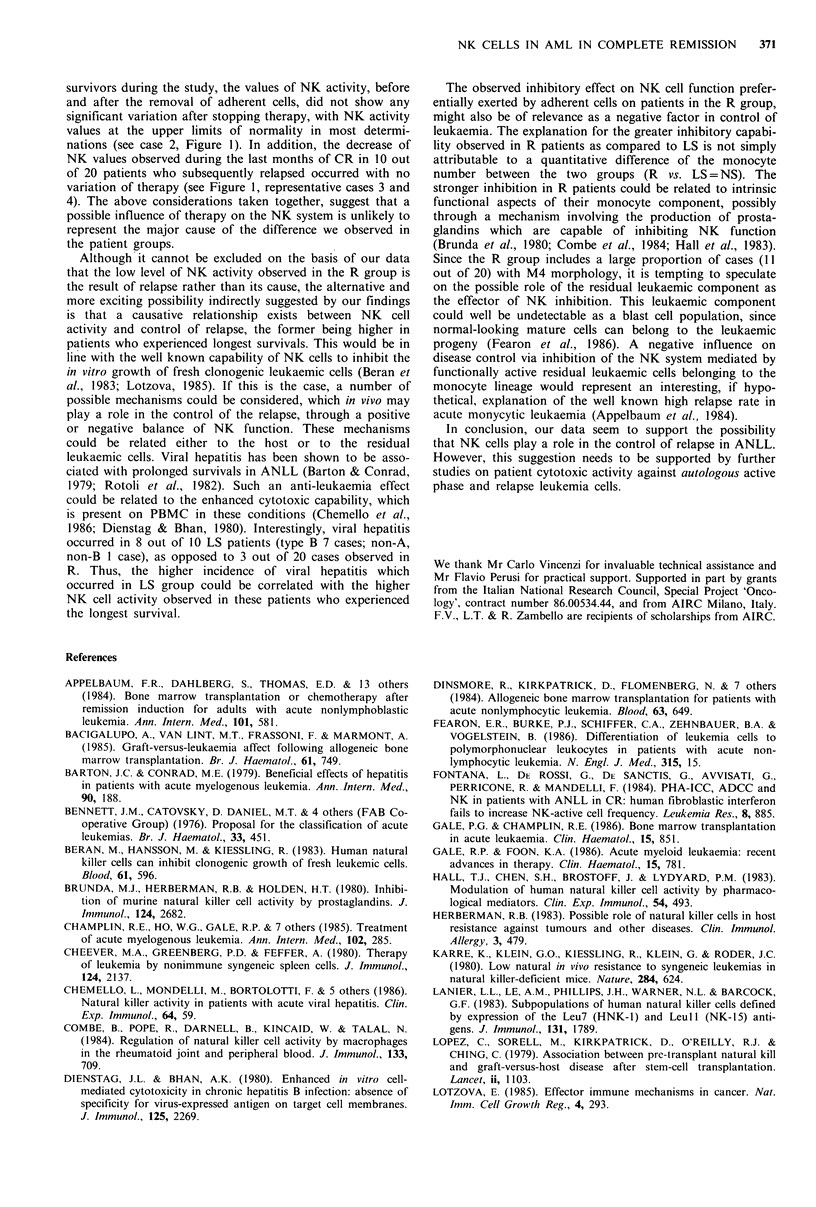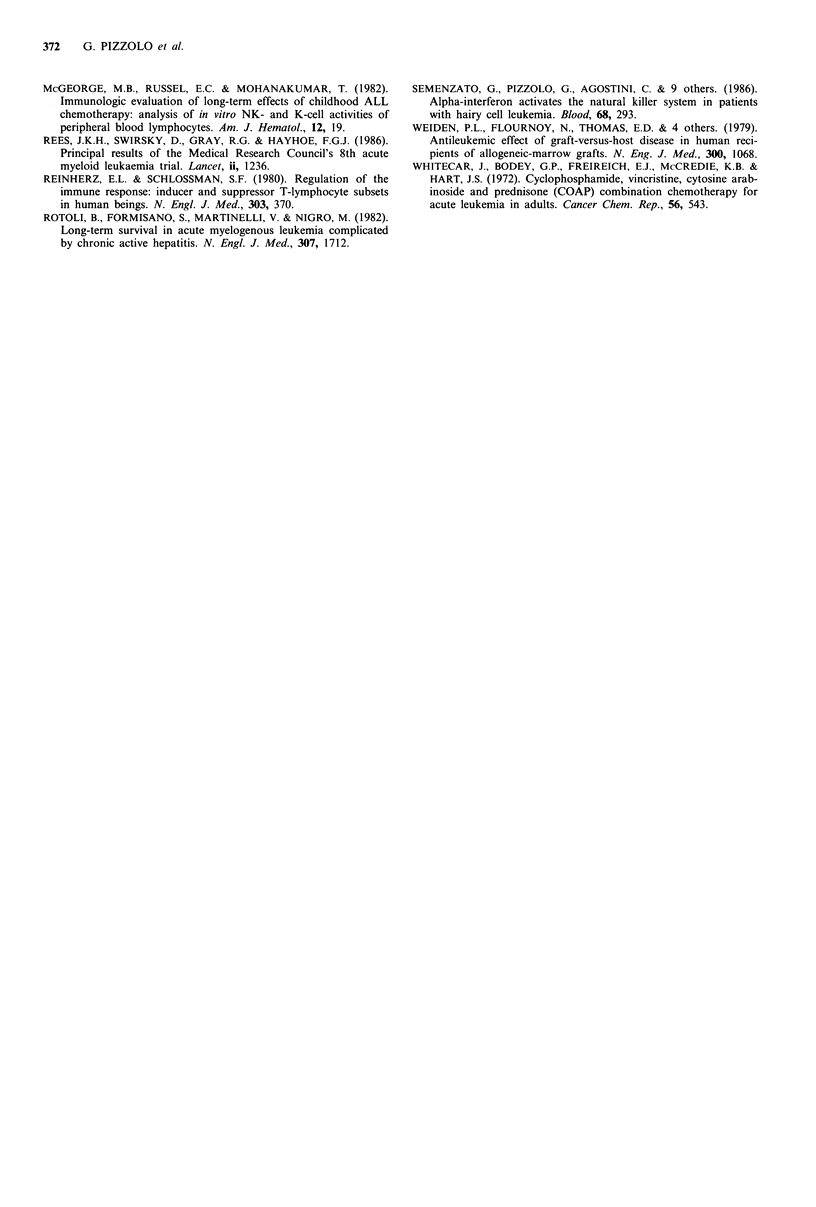# Natural killer cell function and lymphoid subpopulations in acute non-lymphoblastic leukaemia in complete remission.

**DOI:** 10.1038/bjc.1988.221

**Published:** 1988-09

**Authors:** G. Pizzolo, L. Trentin, F. Vinante, C. Agostini, R. Zambello, M. Masciarelli, C. Feruglio, F. Dazzi, G. Todeschini, M. Chilosi

**Affiliations:** Cattedra di Ematologia, University of Verona, Italy.

## Abstract

A long term follow-up study has been undertaken in 33 patients with acute non-lymphoblastic leukaemia (ANLL) in order to establish whether a correlation exists between the clinical course and the immunologic pattern of lymphoid subpopulations. Peripheral blood lymphoid cells have been investigated longitudinally (each 1 to 4 months) during complete remission (CR), by morphologic, phenotypic and functional analyses. Particular attention has been paid to the evaluation of the natural killer (NK) cell compartment, by the detection of cells expressing an NK-related phenotype and by NK in vitro assay. Among the patients so far evaluable, 20 relapsed (R) and 10 are long survivors in CR 'off therapy' (LS). The most relevant finding was represented by statistically higher values of NK activity observed in LS vs. R patients (P less than 0.01). The removal of adherent cells before the NK assay, performed to investigate the possible inhibitory effect on NK function played by the macrophage component, abolished this difference, due to a selective increase of NK function in the R group. The longitudinal study revealed that NK activity tended to decrease in individual patients who subsequently relapsed. These data suggest a possible role of NK cells in the relapse control of ANLL, although it cannot be excluded that the low level of NK activity observed in the R group is the result of impending relapse rather than its cause.


Br. J. Cancer (1988) 58, 368–372
© The Macmillan Press Ltd., 1988

### Natural killer cell function and lymphoid subpopulations in acute non-lymphoblastic leukaemia in complete remission

G. Pizzolo[1], L. Trentin[2], F. Vinante[1], C. Agostini[2], R. Zambello[2], M. Masciarelli[2], C. Feruglio[2], F. Dazzi[1], G. Todeschini[1], M. Chilosi[1], D. Veneri[1], R. Zanotti[1], F. Benedetti[1], G. Perona[1] & G. Semenzato[2]

[1]*Cattedra di Ematologia and Istituto di Anatomia Patologica, University of Verona, Policlinico Borgo Roma, 37134 Verona and* [2]*Istituto di Clinica Medica I and Immunologia Clinica of Padova University, Italy.*

**Summary** A long term follow-up study has been undertaken in 33 patients with acute non-lymphoblastic leukaemia (ANLL) in order to establish whether a correlation exists between the clinical course and the immunologic pattern of lymphoid subpopulations. Peripheral blood lymphoid cells have been investigated longitudinally (each 1 to 4 months) during complete remission (CR), by morphologic, phenotypic and functional analyses. Particular attention has been paid to the evaluation of the natural killer (NK) cell compartment, by the detection of cells expressing an NK-related phenotype and by NK *in vitro* assay. Among the patients so far evaluable, 20 relapsed (R) and 10 are long survivors in CR 'off therapy' (LS). The most relevant finding was represented by statistically higher values of NK activity observed in LS *vs.* R patients ($P<0.01$). The removal of adherent cells before the NK assay, performed to investigate the possible inhibitory effect on NK function played by the macrophage component, abolished this difference, due to a selective increase of NK function in the R group. The longitudinal study revealed that NK activity tended to decrease in individual patients who subsequently relapsed. These data suggest a possible role of NK cells in the relapse control of ANLL, although it cannot be excluded that the low level of NK activity observed in the R group is the result of impending relapse rather than its cause.

Recent advances in the treatment of acute non-lymphoblastic leukaemia (ANLL) led to a substantial increase in the number of patients who experience prolonged complete remissions (CR) which may end in a cure of the disease in a proportion of cases (Champlin *et al.*, 1985). The best results seem to be obtained in patients submitted to allogeneic bone marrow transplantation (BMT) (Dinsmore *et al.*, 1984; Gale & Champlin, 1986). Unfortunately, less than 10% of ANLL patients can take advantage of this procedure for a number of reasons, including the lack of compatible donors, age over 40, and complications related to the transplant (Gale & Champlin, 1986). Despite therapeutic improvements, the overall results seem to indicate that a relapse still occurs within 18 months from the achievement of CR in the majority of patients with ANLL (Champlin *et al.*, 1985; Rees *et al.*, 1986). A number of prognostic factors, possibly independent of treatment, have been suggested to be associated with the duration of CR (reviewed by Gale & Foon, 1986). Some of them are likely to be related to intrinsic biological properties of the leukaemic cells, such as cellular lineage, kinetic parameters, chromosome abnormalities, enzymatic pattern, etc. Other prognostic factors seem to be more connected with the host, including sex, age, and immunologic reactivity. In particular, the last of these could play a role in controlling the proliferation of the residual leukaemic cells eluding the treatment and eventually responsible for the relapse. Beside some experimental evidence (Cheever *et al.*, 1980; Karré *et al.*, 1980), the possibility of such an immunologic control mechanism is also supported by a number of clinical reports. Among them, the beneficial effect of viral hepatitis in ANLL (Barton & Conrad, 1979; Rotoli *et al.*, 1982), and the apparent anti-leukaemia effect of graft-*versus*-host-disease (GvHD) following allogeneic BMT (Weiden *et al.*, 1979; Bacigalupo *et al.*, 1985). Both these conditions are characterised by major alterations of the phenotypic and functional profiles of the reactive lymphoid cells, including those accounting for cytotoxic mechanisms (Chemello *et al.*, 1986; Dienstag & Bhan, 1980; Lopez *et al.*, 1979).

In this paper we investigated the correlations between the clinical course of ANLL and the immunologic pattern of their lymphoid subpopulations, in order to look for indirect evidence of a possible mechanism which might be involved in the control of relapse. For this purpose, peripheral blood mononuclear cells (PBMC) of 33 patients with ANLL in their first CR were longitudinally evaluated by morphologic, phenotypic, and functional approaches. Particular attention was paid to the analysis of the natural killer (NK) cell compartment, since it is believed to represent the first-line of defence against the spread of tumour cells (Herberman, 1983).

Correspondence: G. Pizzolo.
Received 27 February 1988; and in revised form, 25 May 1988.

### Patients and methods

### Patients

Thirty-three patients (15 males, 18 females; mean age 37, range 14–67 years) were studied, from March 1983 to June 1987, during their first CR. All of them had a diagnosis of ANLL made in our institution between January 1978 and December 1986 on the basis of standard criteria, according to the French–American–British (FAB) classification system (Bennett *et al.*, 1976). All patients received a similar treatment based on a standard induction therapy with 3 to 4 courses of adriamycin, thioguanine and cytosine arabinoside, and on a maintenance therapy with COAP regimen (Whitecar *et al.*, 1972) alternated with cytosine arabinoside and thioguanine ($100\,mg\,m^{-2}$ each, twice a day for 5 days) given monthly for 20–30 months after the achievement of CR, or until relapse if it occurred earlier.

Eight out of 33 patients entered the study when they were long survivors (CR > 30 months) 'off therapy' from 3 to 40 months. Seven of them are still in their first CR (CR 65–118 months), and one relapsed. Twenty-five out of 33 patients entered the study during the maintenance therapy, 2 to 20 months from the end of the induction treatment. Among them, 3 are now 'off therapy' long survivors in CR (CR 40, 58, and 65 months), 3 are still on maintenance therapy in CR, and 19 relapsed (CR 4–39 months).

Overall, 10 patients are at present long survivors 'off therapy' in CR (LS group), 20 relapsed (R group), and 3 are still on maintenance therapy not yet long survivors (CR < 30 months). Data on these latter 3 patients have not been considered in the analysis.

### Clinico-haematological evaluation

Clinical reports of all patients were re-evaluated at the time of entry to the study. This was carried out longitudinally during the CR by the concomitant evaluation, at 1 to 4 month intervals, of several clinico-haematological, phenotypic, and functional parameters. In patients on maintenance therapy these multiparameter investigations were performed after a minimum period of 30 days from the end of therapy. The number of observations was 134 in LS and 113 in R, ranging from 3 to 12 in the different patients. Seventy-nine per cent of determinations were performed during maintenance therapy in the R group, as opposed to 19% in LS. The clinico-haematological evaluation included physical examination, complete blood counts and careful observation of May–Grunwald–Giemsa stained PB smears.

*Phenotypic analysis*

Cell suspensions from PBMC were obtained by Ficoll-Hypaque gradient separation from freshly drawn heparinised blood and incubated with fluorochrome-conjugated monoclonal antibodies. These included OKT11 (CD2) (from Ortho Pharmaceutical, Raritan, NJ), Leu3 (CD4), Leu2 (CD8), Leu7, and Leu11 (CD16) (from Becton-Dickinson, Sunnyvale, CA). OKT11 binds the sheep erythrocyte receptor (Reinherz *et al.*, 1980); Leu3 and Leu2 react with the two lymphocyte subsets which include helper and cytotoxic/suppressor cells, respectively (Reinherz *et al.*, 1980); Leu7 and Leu11 react with antigens related to the NK compartment (Lanier *et al.*, 1983). The evaluation was made with a fluorescence microscope on 300 cells per sample.

*NK cell activity*

NK activity was assessed by evaluating the lysis of $^{51}$Cr-labelled K-562 target cells (Semenzato *et al.*, 1986). Briefly, $1 \times 10^6$ target cells were labelled overnight at 37°C in 5% $CO_2$ atmosphere with 100 $\mu$Ci $Na_2(^{51}Cr)O_4$ (CIA IRE Sorin, Biomedica, Saluggia, Italy) and extensively washed before use. Cells ($1 \times 10^4$) were resuspended in each well of a V-shaped plate (Titertech, Falcon Lab.) and graded concentrations of PBMC were added to wells in triplicate and incubated for 4 h at 37°C in 5% $CO_2$. Following this incubation, supernatants were harvested and counted in a gamma counter. The mean value of triplicate assays was used to calculate the percentage of cytotoxicity, as already reported. The analysis of NK activity was performed comparing the values of cytotoxicity in the different groups at an effector/target (E/T) ratio of 40:1. In 74% of determinations NK activity was carried out simultaneously on unfractionated PBMC and after removal of monocytes by adherence to plastic dishes, as previously reported in detail (Semenzato *et al.*, 1986).

*Statistical analysis*

The number of determinations for each parameter was variable from patient to patient, depending on the follow-up period. Therefore, in order to avoid a disproportionate effect of patients tested on a larger number of occasions, the mean values were not calculated on the basis of each test result but using one value per patient which represented the mean of all figures observed in that patient. Results are expressed as mean values ± standard deviation (s.d.) and the comparison between values has been performed using the Student's *t*-test and analysis of variance (ANOVA).

### Results

*Clinico-haematological evaluation*

The age and sex distribution was similar in the two groups. The various morphological categories of the FAB classification were nearly all represented in both groups, with a higher concentration of M4 (myelo-monocytic type) in R (11 out of 20 cases) as compared to LS (2/10). The presence of antibodies against virus hepatitis B (HBsAb+) was detected in 7 out of 10 cases (70%) in LS, *versus* 3 out of 20 (15%) in R. In most HBsAb+ cases, clinical and/or laboratory signs of hepatitis had been observed during the induction treatment or soon after the achievement of CR. Furthermore, one patient belonging to the LS group had developed a non-A, non-B viral hepatitis.

The absolute numbers (mean ± s.d.) of lymphocytes, monocytes, and large granular lymphocytes (LGL), as emerged from all longitudinal determinations, are shown in Table I. The mean values of lymphocytes were similar to age-matched controls in both groups. The monocyte values were similar in LS and R, but the figures were statistically different from controls only in LS (LS<C; $P<0.01$). LGL values were higher in LS as compared to R ($P<0.05$) and controls ($P<0.01$).

*Phenotypic analysis*

Data are also shown in Table I. The mean values of T cells (CD2+) in LS and R were similar to controls. The CD4/CD8 ratio was lower than control values both in LS and R. Leu11+ cells were higher in LS than in R ($P<0.05$). The mean values of Leu7+ cells, although slightly higher in LS as compared to R, were not statistically different in the two groups.

*NK cell activity*

The overall results of NK activity detected on PBMC non-monocyte-depleted at E/T ratio of 40:1 are reported in Table II. The figures were higher in LS as compared to R ($P<0.01$). However, the LS values were not statistically different from controls. The analysis of individual values indicates that determinations below the interval of normality were 18% in LS as compared to 57% in R. On the other hand, the determinations exceeding the control range were 31% in LS, and 5% in R. Monocyte depletion by the removal of adherent cells reduced the differences in terms of NK activity observed between LS and R on PBMC 'non-depleted' (Table II). This was mainly due to an increase of

**Table I** Morphologic and phenotypic analyses of lymphoid cells in ANLL in CR. Mean values ± s.d. of longitudinal data in long survivors, patients who subsequently relapsed, and in controls.

| *Parameters* | *Long survivors* (*LS = 10*) | *Relapsed* (*R = 20*) | *Controls* (*C = 25*) | P *value* |
|---|---|---|---|---|
| Lymphocytes[a] | 2.02 ± 0.44 | 2.02 ± 0.66 | 2.32 ± 0.49 | NS |
| LGL[a] | 0.75 ± 0.33 | 0.48 ± 0.26 | 0.36 ± 0.17 | LS − C < 0.01<br>LS − R < 0.05 |
| Monocytes[a] | 0.31 ± 0.08 | 0.34 ± 0.15 | 0.42 ± 0.11 | LS − C < 0.01 |
| CD2+ (T11+)[a] | 1.35 ± 0.34 | 1.33 ± 0.49 | 1.67 ± 0.27 | R − C < 0.01 |
| Leu7+[a] | 0.41 ± 0.22 | 0.32 ± 0.18 | 0.44 ± 0.24 | NS |
| Leu11+[a] | 0.23 ± 0.15 | 0.13 ± 0.09 | 0.18 ± 0.12 | LS − R < 0.05 |
| CD4/CD8 | 1.62 ± 0.73 | 1.51 ± 0.87 | 2.40 ± 0.79 | R − C < 0.01 |

[a] $\times 10^9 l^{-1}$; NS: not significant.

**Table II** *In vitro* NK activity of PBMC (as expressed by percentage of $^{51}Cr$ release at effector target ratio 40:1) in patients with ANLL in CR and controls (mean values ± s.d.)

| *Patient groups* | *Percentage of $^{51}Cr$ release* — *PBMC non-depleted* | *PBMC monocyte depleted* | P *value* |
|---|---|---|---|
| | A | B | A *vs.* B |
| Long survivors (LS) | 36.0 ± 10.3 | 39.6 ± 10.6 | NS |
| Relapsed (R) | 19.7 ± 11.4 | 28.3 ± 11.7 | $P<0.001$ |
| Controls (C) | 32.3 ± 11.0 | 34.2 ± 13.4 | NS |
| *P* value: LS *vs.* R: | $P<0.01$ | NS | |
| LS *vs.* C: | NS | NS | |
| R *vs.* C: | $P<0.02$ | NS | |

NK activity in R (from 19.7 ± 11.4 to 28.3 ± 11.7; $P<0.001$). NK values before and after monocyte removal were not statistically different in LS and C.

*Phenotypic and functional fluctuation during CR*

In the majority of patients the various lymphoid subpopulations, as defined by morphologic and phenotypic parameters, did not show major fluctuations during the observation period. However, in 12 out of 30 cases this was found to occur for one or more of the considered parameters. Although in these patients it was difficult to establish a definite trend, in 9 cases who subsequently relapsed a decrease of Leu7, Leu11, and CD4/CD8 ratio values could be observed 2 to 4 months prior to the relapse.

The data on NK cell activity analysed in individual patients showed a more composite pattern. In LS, 4 out of 10 patients had values consistently exceeding the range of controls (Figure 1, representative case 1), 4 had the large majority of their values within the normal range (Figure 1, representative case 2) and 2 showed wide fluctuations with several values below the normal range. Among the 20 patients of the R group, the values during the follow-up period were mostly concentrated below or at the lower limit of normality in 14 cases (Figure 1, representative case 4) while they were variable and scattered in 6. In 10 cases of this group (see Figure 1, representative case 3) the NK values observed during the last 1–4 months of CR, just before the relapse, dropped to lower levels as compared to the mean values of previous determinations of each given case. In these patients the reduction of $^{51}Cr$ release of pre-relapse values was between 9 and 30% (mean 17%).

### Discussion

Our study indicates that long surviving patients with ANLL in CR exhibit higher *in vitro* NK activity of PBMC, associated with increased levels of NK-related cells (LGL and Leu11+), as compared to patients who subsequently relapsed. Other major phenotypic differences were not observed between the two groups. These data suggest a possible role of NK cells in the relapse control of ANLL. However, this suggestion has to be considered with caution, since the study is also open to other plausible interpretations. First, the differences observed between the two groups could be related to the effect of therapy. In fact, 79% of determinations were performed during maintenance therapy in the R group, as opposed to 19% in LS. Although it is possible that therapy might have influenced some parameters, a number of considerations suggest that the differences observed in terms of NK function and/or inhibition of NK activity by adherent cells, may not be merely attributable to therapy. The therapy-related depression of NK function previously reported in patients with acute leukaemia (Fontana *et al.*, 1984; McGeorge *et al.*, 1982) was shown to be confined to the period of treatment, with recovery of NK activity within days after therapy ceased (McGeorge *et al.*, 1982). In all patients investigated during maintenance therapy, immunological tests were performed just prior to initiating each subsequent course, at least one month after the previous one. Other indirect suggestions against a major role of therapy in modifying the results come from the analysis of data in individual patients. In 3 cases who became long-term

**Figure 1** *In vitro* longitudinal variations of NK activity of PBMC in 4 representative cases of patients with ANLL investigated during complete remission. Shaded area defines the range (mean ± s.d.) of controls. Months refer to the achievement of complete remission. Arrows in cases 3 and 4 indicate the time of relapse.

survivors during the study, the values of NK activity, before and after the removal of adherent cells, did not show any significant variation after stopping therapy, with NK activity values at the upper limits of normality in most determinations (see case 2, Figure 1). In addition, the decrease of NK values observed during the last months of CR in 10 out of 20 patients who subsequently relapsed occurred with no variation of therapy (see Figure 1, representative cases 3 and 4). The above considerations taken together, suggest that a possible influence of therapy on the NK system is unlikely to represent the major cause of the difference we observed in the patient groups.

Although it cannot be excluded on the basis of our data that the low level of NK activity observed in the R group is the result of relapse rather than its cause, the alternative and more exciting possibility indirectly suggested by our findings is that a causative relationship exists between NK cell activity and control of relapse, the former being higher in patients who experienced longest survivals. This would be in line with the well known capability of NK cells to inhibit the *in vitro* growth of fresh clonogenic leukaemic cells (Beran *et al.*, 1983; Lotzova, 1985). If this is the case, a number of possible mechanisms could be considered, which *in vivo* may play a role in the control of the relapse, through a positive or negative balance of NK function. These mechanisms could be related either to the host or to the residual leukaemic cells. Viral hepatitis has been shown to be associated with prolonged survivals in ANLL (Barton & Conrad, 1979; Rotoli *et al.*, 1982). Such an anti-leukaemia effect could be related to the enhanced cytotoxic capability, which is present on PBMC in these conditions (Chemello *et al.*, 1986; Dienstag & Bhan, 1980). Interestingly, viral hepatitis occurred in 8 out of 10 LS patients (type B 7 cases; non-A, non-B 1 case), as opposed to 3 out of 20 cases observed in R. Thus, the higher incidence of viral hepatitis which occurred in LS group could be correlated with the higher NK cell activity observed in these patients who experienced the longest survival.

The observed inhibitory effect on NK cell function preferentially exerted by adherent cells on patients in the R group, might also be of relevance as a negative factor in control of leukaemia. The explanation for the greater inhibitory capability observed in R patients as compared to LS is not simply attributable to a quantitative difference of the monocyte number between the two groups (R *vs.* LS = NS). The stronger inhibition in R patients could be related to intrinsic functional aspects of their monocyte component, possibly through a mechanism involving the production of prostaglandins which are capable of inhibiting NK function (Brunda *et al.*, 1980; Combe *et al.*, 1984; Hall *et al.*, 1983). Since the R group includes a large proportion of cases (11 out of 20) with M4 morphology, it is tempting to speculate on the possible role of the residual leukaemic component as the effector of NK inhibition. This leukaemic component could well be undetectable as a blast cell population, since normal-looking mature cells can belong to the leukaemic progeny (Fearon *et al.*, 1986). A negative influence on disease control via inhibition of the NK system mediated by functionally active residual leukaemic cells belonging to the monocyte lineage would represent an interesting, if hypothetical, explanation of the well known high relapse rate in acute monycytic leukaemia (Appelbaum *et al.*, 1984).

In conclusion, our data seem to support the possibility that NK cells play a role in the control of relapse in ANLL. However, this suggestion needs to be supported by further studies on patient cytotoxic activity against *autologous* active phase and relapse leukemia cells.

We thank Mr Carlo Vincenzi for invaluable technical assistance and Mr Flavio Perusi for practical support. Supported in part by grants from the Italian National Research Council, Special Project 'Oncology', contract number 86.00534.44, and from AIRC Milano, Italy. F.V., L.T. & R. Zambello are recipients of scholarships from AIRC.

### References

APPELBAUM, F.R., DAHLBERG, S., THOMAS, E.D. & 13 others (1984). Bone marrow transplantation or chemotherapy after remission induction for adults with acute nonlymphoblastic leukemia. *Ann. Intern. Med.*, **101**, 581.

BACIGALUPO, A., VAN LINT, M.T., FRASSONI, F. & MARMONT, A. (1985). Graft-versus-leukaemia affect following allogeneic bone marrow transplantation. *Br. J. Haematol.*, **61**, 749.

BARTON, J.C. & CONRAD, M.E. (1979). Beneficial effects of hepatitis in patients with acute myelogenous leukemia. *Ann. Intern. Med.*, **90**, 188.

BENNETT, J.M., CATOVSKY, D. DANIEL, M.T. & 4 others (FAB Cooperative Group) (1976). Proposal for the classification of acute leukemias. *Br. J. Haematol.*, **33**, 451.

BERAN, M., HANSSON, M. & KIESSLING, R. (1983). Human natural killer cells can inhibit clonogenic growth of fresh leukemic cells. *Blood*, **61**, 596.

BRUNDA, M.J., HERBERMAN, R.B. & HOLDEN, H.T. (1980). Inhibition of murine natural killer cell activity by prostaglandins. *J. Immunol.*, **124**, 2682.

CHAMPLIN, R.E., HO, W.G., GALE, R.P. & 7 others (1985). Treatment of acute myelogenous leukemia. *Ann. Intern. Med.*, **102**, 285.

CHEEVER, M.A., GREENBERG, P.D. & FEFFER, A. (1980). Therapy of leukemia by nonimmune syngeneic spleen cells. *J. Immunol.*, **124**, 2137.

CHEMELLO, L., MONDELLI, M., BORTOLOTTI, F. & 5 others (1986). Natural killer activity in patients with acute viral hepatitis. *Clin. Exp. Immunol.*, **64**, 59.

COMBE, B., POPE, R., DARNELL, B., KINCAID, W. & TALAL, N. (1984). Regulation of natural killer cell activity by macrophages in the rheumatoid joint and peripheral blood. *J. Immunol.*, **133**, 709.

DIENSTAG, J.L. & BHAN, A.K. (1980). Enhanced *in vitro* cell-mediated cytotoxicity in chronic hepatitis B infection: absence of specificity for virus-expressed antigen on target cell membranes. *J. Immunol.*, **125**, 2269.

DINSMORE, R., KIRKPATRICK, D., FLOMENBERG, N. & 7 others (1984). Allogeneic bone marrow transplantation for patients with acute nonlymphocytic leukemia. *Blood*, **63**, 649.

FEARON, E.R., BURKE, P.J., SCHIFFER, C.A., ZEHNBAUER, B.A. & VOGELSTEIN, B. (1986). Differentiation of leukemia cells to polymorphonuclear leukocytes in patients with acute nonlymphocytic leukemia. *N. Engl. J. Med.*, **315**, 15.

FONTANA, L., DE ROSSI, G., DE SANCTIS, G., AVVISATI, G., PERRICONE, R. & MANDELLI, F. (1984). PHA-ICC, ADCC and NK in patients with ANLL in CR: human fibroblastic interferon fails to increase NK-active cell frequency. *Leukemia Res.*, **8**, 885.

GALE, P.G. & CHAMPLIN, R.E. (1986). Bone marrow transplantation in acute leukaemia. *Clin. Haematol.*, **15**, 851.

GALE, R.P. & FOON, K.A. (1986). Acute myeloid leukaemia: recent advances in therapy. *Clin. Haematol.*, **15**, 781.

HALL, T.J., CHEN, S.H., BROSTOFF, J. & LYDYARD, P.M. (1983). Modulation of human natural killer cell activity by pharmacological mediators. *Clin. Exp. Immunol.*, **54**, 493.

HERBERMAN, R.B. (1983). Possible role of natural killer cells in host resistance against tumours and other diseases. *Clin. Immunol. Allergy*, **3**, 479.

KARRE, K., KLEIN, G.O., KIESSLING, R., KLEIN, G. & RODER, J.C. (1980). Low natural *in vivo* resistance to syngeneic leukemias in natural killer-deficient mice. *Nature*, **284**, 624.

LANIER, L.L., LE, A.M., PHILLIPS, J.H., WARNER, N.L. & BARCOCK, G.F. (1983). Subpopulations of human natural killer cells defined by expression of the Leu7 (HNK-1) and Leu11 (NK-15) antigens. *J. Immunol.*, **131**, 1789.

LOPEZ, C., SORELL, M., KIRKPATRICK, D., O'REILLY, R.J. & CHING, C. (1979). Association between pre-transplant natural kill and graft-versus-host disease after stem-cell transplantation. *Lancet*, **ii**, 1103.

LOTZOVA, E. (1985). Effector immune mechanisms in cancer. *Nat. Imm. Cell Growth Reg.*, **4**, 293.

McGEORGE, M.B., RUSSEL, E.C. & MOHANAKUMAR, T. (1982). Immunologic evaluation of long-term effects of childhood ALL chemotherapy: analysis of *in vitro* NK- and K-cell activities of peripheral blood lymphocytes. *Am. J. Hematol.*, **12**, 19.

REES, J.K.H., SWIRSKY, D., GRAY, R.G. & HAYHOE, F.G.J. (1986). Principal results of the Medical Research Council's 8th acute myeloid leukaemia trial. *Lancet*, **ii**, 1236.

REINHERZ, E.L. & SCHLOSSMAN, S.F. (1980). Regulation of the immune response: inducer and suppressor T-lymphocyte subsets in human beings. *N. Engl. J. Med.*, **303**, 370.

ROTOLI, B., FORMISANO, S., MARTINELLI, V. & NIGRO, M. (1982). Long-term survival in acute myelogenous leukemia complicated by chronic active hepatitis. *N. Engl. J. Med.*, **307**, 1712.

SEMENZATO, G., PIZZOLO, G., AGOSTINI, C. & 9 others. (1986). Alpha-interferon activates the natural killer system in patients with hairy cell leukemia. *Blood*, **68**, 293.

WEIDEN, P.L., FLOURNOY, N., THOMAS, E.D. & 4 others. (1979). Antileukemic effect of graft-versus-host disease in human recipients of allogeneic-marrow grafts. *N. Eng. J. Med.*, **300**, 1068.

WHITECAR, J., BODEY, G.P., FREIREICH, E.J., McCREDIE, K.B. & HART, J.S. (1972). Cyclophosphamide, vincristine, cytosine arabinoside and prednisone (COAP) combination chemotherapy for acute leukemia in adults. *Cancer Chem. Rep.*, **56**, 543.